# Evaluating the predictive capacity of coral reef resilience assessments

**DOI:** 10.1038/s41598-026-52791-2

**Published:** 2026-05-30

**Authors:** Orion S. McCarthy, Eric Conklin, Katharine Ricke, Russell T. Sparks, Jennifer E. Smith

**Affiliations:** 1 Scripps Institution of Oceanography, UC San Diego, La Jolla, San Diego, CA 92037 USA; 2 The Nature Conservancy – Hawai’i and Palmyra, Honolulu, HI 96817 USA; 3 School of Global Policy and Strategy, UC San Diego, La Jolla, San Diego, 92037 CA USA; 4 Hawaii Division of Aquatic Resources (Maui Office), Department of Land and Natural Resources, Wailuku, HI 96793 USA

**Keywords:** Climate sciences, Ecology, Ecology, Ocean sciences, Scientific community

## Abstract

Resilience assessments, which rely on ecological indicators to predict future ecosystem resilience across a management jurisdiction, are used to prioritize limited conservation resources toward “resilient” locations with the best chance of surviving climate change. While resilience assessments have been widely applied to inform management, particularly for coral reefs, their precision and accuracy have rarely been validated. We used a timeseries of coral reef 3D models to (1) conduct multiple resilience assessments of fixed sites over time, and (2) track the resistance and recovery of those same sites over a decade. This allowed us to compare resilience predictions across assessments (precision) and test whether resilience predictions aligned with observed patterns of resistance and recovery (accuracy). We found that resilience assessments are capable of generating consistent resilience predictions over time, but in our case those predictions were not correlated with observed resistance or recovery dynamics. We recommend modelling resistance and recovery as distinct processes, and emphasize the importance of validating predictions with long-term monitoring. We caution against using resilience assessments in isolation to drive conservation decision making. Instead, spatial prioritization should be driven by local community input, with management of priority sites informed by functional indicators of resistance and recovery.

**Supplementary Information:**

The online version contains supplementary material available at 10.1038/s41598-026-52791-2.

## Introduction

Natural resource managers often grapple with limited financial or political resources, necessitating strategic consideration of where, when, and how to deploy resources for conservation^1,2^. This need for triage has fueled the use of ecological indicators to predict ecosystem change, prioritize locations for conservation, and measure progress toward management goals at priority sites^3^. These efforts range from ecosystem scorecards tailored to a specific management context^4,5^, to global efforts to develop standardized biodiversity indicators to underpin emerging nature markets and global stocktaking efforts^6–8^. Regardless of the specific application, the utility of these indicator sets depends on the ecological relevance of the indicators selected, as well as the assumptions made when combining indicators into a synthetic index, ranking, or prediction^3,8–10^. If applied inappropriately, these approaches risk misdirecting limited conservation resources.

One widely-used application of ecological indicators is for resilience-based management, a spatial prioritization approach that seeks to ensure the persistence of “resilient” locations through site-specific management interventions^11,12^. In this context, “resilient” locations have stable ecological communities that demonstrate the capacity for both resistance (persistence through disturbance events without undergoing major turnover) and recovery (the ability to return to pre-disturbance condition without shifting to a less desirable ecosystem state)^13,14^. Many resilience-based management applications explicitly focus on climate-driven disturbances, where locations with high resilience and low exposure to disturbance (e.g., climate refugia) are considered to have lower vulnerability^15,16^. In practice, these locations are identified by monitoring ecological indicators across multiple sites. The exact suite of ecological indicators depends on the ecosystem, but typically includes metrics of habitat structure (e.g., percent cover of foundation species), composition (e.g., species richness), and function (e.g., recruitment, competition, herbivory)^14^. This monitoring data is used to predict each site’s vulnerability, often in concert with anthropogenic and/or social indicators^17^. This process is known as a “resilience assessment”. Most commonly, this approach prioritizes management interventions at sites with low vulnerability, following the assumption that efforts to reduce local human impacts at resilient locations will maximize the likelihood that at least some locations persist under climate change^15,18^.

Resilience-based management has particular salience for coral reef conservation, given the immediate severity of climate threats faced by reefs globally^19,20^. In addition to climate change, much of the world’s coral reefs also face localized threats from natural and anthropogenic stressors including overfishing, coastal development, nutrient pollution, sedimentation, tourism, and outbreaks of coral disease and crown of thorns starfish^21^. From a conservation perspective, it is imperative to address both local and global stressors to ensure continued coral reef functioning in the 21st century^2^. International action is required to reduce greenhouse gas emissions and prevent worst-case scenarios for coral reefs globally^19^. However, in the absence of global climate action, managers must focus on mitigating local threats to reefs in their jurisdiction, such as by imposing fishing regulations, establishing marine protected areas, or reducing urban runoff^12,22–25^.

The concept of resilience-based management was first proposed for coral reefs more than two decades ago^26^ and began to be operationalized via resilience assessments in the early 2010s^14,27,28^. Coral reef resilience assessments have since been conducted in more than 40 countries and territories^12^, spanning the Indian Ocean^29–31^, Coral Triangle^18,32,33^, Pacific^17,34–37^, and Caribbean^38–41^. These assessments have been largely successful at informing site-specific management actions, due in part to the active participation of non-governmental organizations and government agencies^12^.

Despite the growing popularity of resilience-based management, a number of uncertainties exist about its efficacy and equity. In practice, most resilience assessments are conducted at a single point in time^12,35^, and few studies exist that test the accuracy of resilience assessment predictions following major climate-driven disturbance events^37,42^. Furthermore, the relative importance of different ecological indicators, as well as their functional relationship to resilience, is not fully understood^11,14,37,43^. As a result, it is unclear how sensitive resilience assessments are to the choice of indicators or to their weighting, the methods used to assess indicators, or the year in which the assessment is conducted. Answers to these questions would help clarify the efficacy of resilience assessments as currently implemented, and could identify potential adjustments to improve their precision and accuracy.

These knowledge gaps create the potential for adverse outcomes, especially when resilience assessments are used prescriptively or applied in isolation to prioritize locations for management. For example, if resilience predictions conflict with community priorities, managers are faced with the prospect of directing limited conservation resources away from locations that are culturally or socially significant to their constituency. While this may be warranted under specific circumstances, confidence in resilience predictions would need to be high, and the ecological objectives dire, to justify such prioritization. Marine spatial planning is inherently complex, and top-down approaches that do not account for the socio-cultural objectives of multiple stakeholder groups will have lower rates of success^44^. Uncertainty around resilience assessment efficacy has potential to undermine community support for conservation and misdirect limited resources.

A rigorous evaluation of resilience assessment predictive capacity of is overdue. In this study, we used a decade-long timeseries of large-area imagery (3D coral reef models reconstructed via photogrammetry) to conduct multiple sequential resilience assessments of fixed sites in leeward Maui. We compared resilience predictions made with large-area imagery to resilience predictions made by Maynard et al. 2019^36^, a resilience assessment conducted using in situ monitoring surveys from leeward Maui in 2018. Our study represents the first time large-area imagery has been used to conduct a resilience assessment^45^. In addition, our large-area imagery timeseries allowed us to observe patterns of benthic community resistance and recovery with respect to a major bleaching event, which occurred one year after our timeseries began (the large-area imagery spans 2014 to 2024, and bleaching occurred in 2015). This study design allowed us to answer the following questions: (1) are site-level predictions of relative resilience robust to differences in methodology and survey year; and (2) do resilience assessments accurately predict resistance and recovery over time?

## Results and discussion

To be effective, resilience assessments must be both precise and accurate: they should consistently prioritize the same locations regardless of the sampling methodology used, and they should accurately predict ecosystem resistance and recovery potential. In this study, we evaluated resilience assessment precision by comparing the predictions of six resilience assessments conducted at the same fixed sites in leeward Maui. Each assessment used the procedures of Maynard et al. 2019^36^ to rank sites based on their relative resilience according to a standard set of indicators (Table 1), but each assessment relied on different input data (large-area imagery surveys from 2014, 2015, 2017, 2019, and 2021, and in situ surveys from 2018^36^. Once we predicted resilience, we evaluated assessment accuracy by comparing those predictions with actual patterns of reef change over time, focusing on evidence of resistance (persistence through disturbance events without undergoing major turnover) and recovery (the ability to return to pre-disturbance condition without shifting to a less desirable ecosystem state).

We used our large-area imagery timeseries to quantify processes associated with resistance and recovery at both the community and colony scales (Table 2). At the community scale, we used benthic percent cover data to calculate change in coral cover and Bray-Curtis similarity over time. At the colony scale, we assessed colony condition and delineated colony boundaries through time to derive site-level rates of growth, survivorship, thermal tolerance, and acclimatization^46^, along with colony size frequency distributions. These data allowed us to quantify patterns of benthic succession over a decade as well as the population demographics of four key reef-building taxa. This multi-scale approach enabled us to account for different processes underlying resistance and recovery that may only be evident at a given range of scales^47^. For example, a shift from fleshy to calcifying algae dominance would indicate important shifts in nutrient input or herbivory^48^ and would be more readily detected at the community scale. Conversely, a pulse in coral recruitment would indicate population fecundity and future coral growth, and would be more readily detected at the colony scale^49^. Importantly, all of our metrics of resistance and recovery (at both scales) were integrated over time, which contrasts to the indicators used in the resilience assessment (Table 1), which were measured at a single point in time.

**Table 1:** A description of the ecological indicators used to conduct resilience assessments in leeward Maui, adapted from Maynard et al. 2019^36^.

Indicator	Description	Functional linkage to resilience	Survey method (in situ)	Survey method (3D timeseries)
Coral cover	Percent of the benthos (%) occupied by living corals.	Corals provide many of a reef’s ecosystem services, such as habitat provisioning and wave attenuation. Higher coral cover is generally seen as a positive indicator of reef health.	Percent cover was assessed using 20 randomly selected photoquadrats (0.8 × 0.6 m, 30 random points per photo) collected along three 25 m transects in 2018.	Percent cover was assessed by identifying benthic taxa at 2,500 stratified random points across a single virtual quadrat (10 × 10 m). Measured annually (2014–2024).
Reef builder ratio	Ratio of the percent cover of corals and calcifying algae to the percent cover of fleshy algae and other sessile invertebrates.	Corals and calcifying algae cement the reef together and provide settlement substrate for coral recruits. Reef builder ratio represents the balance between these calcifying organisms and non-calcifiers.	Derived from percent cover survey in 2018.	Derived from annual percent cover surveys (2014–2024).
Coral diversity	Simpsons diversity index for coral community (unitless, ranges from 0 to 1).	A more diverse assemblage of corals has higher functional redundancy and can provide more habitat niches for different reef organisms.	Derived from percent cover survey in 2018.	Derived from annual percent cover surveys (2014–2024).
Coral health	Percent (%) of colonies surveyed with signs of disease, bleaching, or algal overgrowth.	Indicators of compromised coral health, such as disease, bleaching, and algal overgrowth, are signs that corals are facing chronic stress.	Visual survey of coral health within twelve 0.25 m^2^ quadrats in 2018.	Virtual survey of coral health within ten 0.5 m^2^ quadrats. Measured in 2014, 2015, 2017, 2019, and 2021.
Juvenile coral density	Density (#/m²) of colonies under 5 cm in diameter.	Coral recruits represent a demographic influx of new individuals into the population. Coral recruitment is a mechanism for coral recovery after disturbance.	Visual survey of coral juveniles (all species) within twelve 0.25 m^2^ quadrats in 2018.	Virtual survey of coral juveniles (*Pocillopora* only) within a single 12 × 12 m quadrat. Measured in 2014, 2015, 2017, 2019, and 2021.
Linear rugosity	Ratio of the distance traversed by the reef’s contour to the distance traversed by a straight line. (unitless, lower bounded by 1).	Rugosity is a metric of habitat structural complexity. Habitats with more structural complexity can support a more diverse assemblage of fish and invertebrates.	Measured the reef contour using a 10 m brass chain at the start each percent cover transect in 2018.	Measured the reef contour within a 10 × 10 m quadrat using 99 virtual profile gauges. Measured annually (2014–2024).
Herbivorous fish biomass	The biomass (g/m²) of all herbivorous fish.	Herbivorous fish eat algae that can overgrow and outcompete corals. Grazing by these fish help to maintain balance between corals and algae on the reef.	Estimated the length and biomass of herbivorous fish within three 25 × 5 m belt transects in 2018.	Not measured.

Survey methods are briefly summarized for each indicator, both for the 2018 in situ assessment and for the 3D model timeseries used in this study. For this study, we omitted herbivorous fish biomass because that data was not captured in the large-area imagery timeseries.

**Table 2:** Metrics used to quantify patterns of resistance (persistence through disturbance events without undergoing major turnover) and recovery (the ability to return to pre-disturbance condition without shifting to a less desirable ecosystem state) observed in relation to a major bleaching event (2015).

	Scale	Metric	Description	Timeframe
Resistance	Community	Bray-Curtis Similarity	Similarity of pre- and post-bleaching benthic community (high similarity = high resistance)	2015–2016 (1 year)
	Community	Coral cover	Percent of pre-bleaching coral cover lost (low coral cover loss = high resistance)	2015–2016 (1 year)
	Colony	Size frequency distribution	Shift in colony size frequency distribution (no shift toward smaller colonies = high resistance)	2014–2017 (3 years)
	Colony*	Bleaching susceptibility	Proportion of colonies that exhibited signs of thermal tolerance or acclimatization between 2015 and 2019 bleaching events (more thermally tolerant or acclimatized colonies = high resistance)	2014–2021 (7 years)
	Colony*	Coral survivorship	Probability of colony survival as a function of initial colony size (high survivorship = high resistance)	2014–2021 (7 years)
Recovery	Community	Bray-Curtis Similarity	Annual rate of change in similarity between pre- and post-bleaching communities (increasing similarity to pre-bleaching community over time = high recovery)	2016–2024 (8 years)
	Community	Coral cover	Percent of pre-bleaching coral cover regained annually (increasing coral cover over time = high recovery)	2016–2024 (8 years)
	Colony	Size frequency distribution	Shift in colony size frequency distribution (shift toward pre-bleaching distribution = high recovery)	2017–2021 (4 years)
	Colony*	Coral growth	Change in colony area (more coral growth = high recovery)	2014–2021 (7 years)

Multiple metrics were used to capture resistance and recovery dynamics observed at different scales (community scale and colony scale). Patterns of bleaching susceptibility, coral growth, and coral survivorship are sourced from McCarthy et al. 2024^46^. (denoted by *).

Overall, we found that resilience assessments are capable of producing reasonably precise rankings of relative resilience, but that these rankings did not accurately predict the resistance and recovery trends we observed at our sites in leeward Maui.

### High precision of resilience assessment predictions

**Fig. 1:**
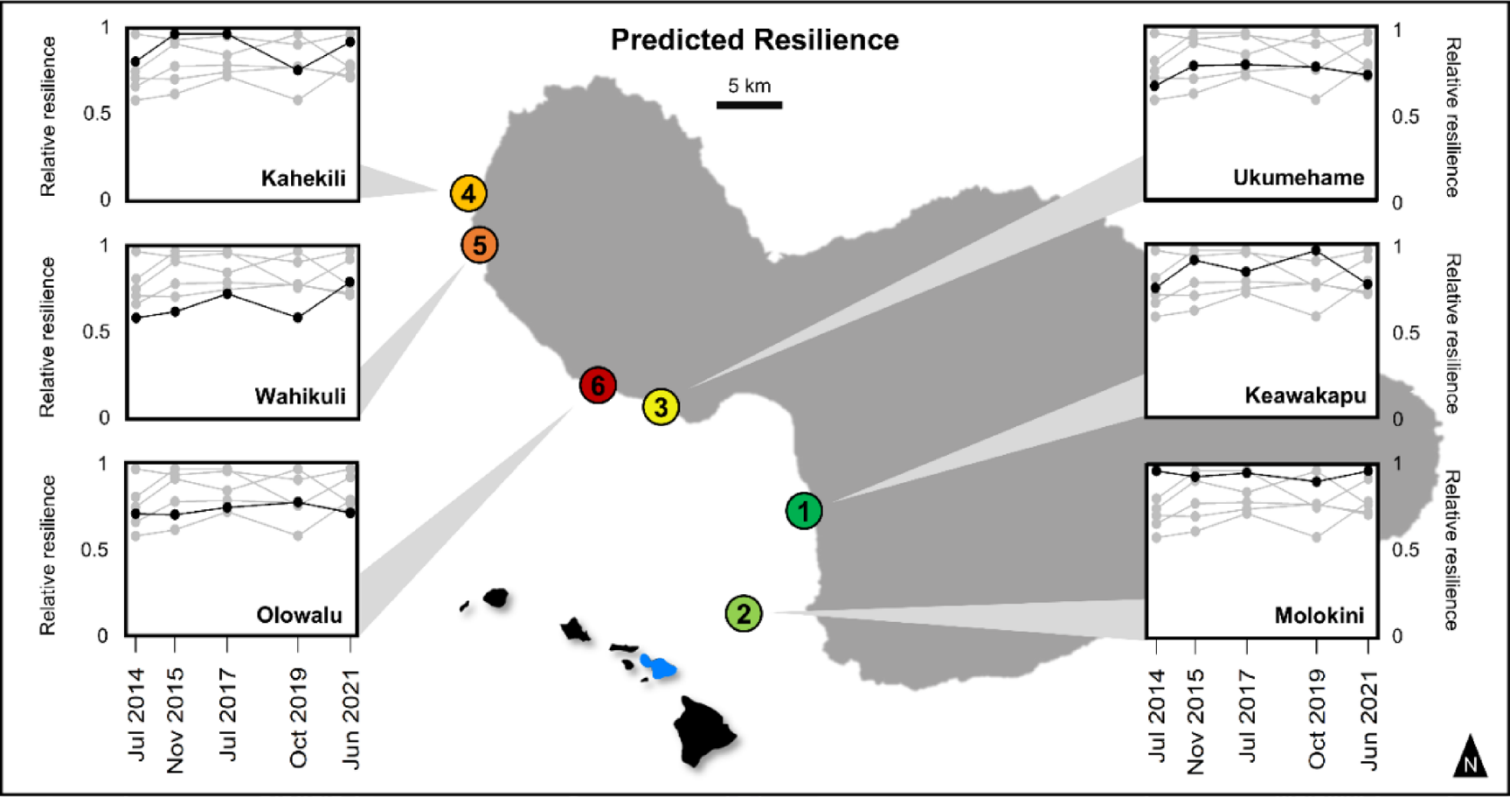
A map of Maui showing the six resilience assessment sites in this study, symbolized by the resilience predictions of the 2018 in situ resilience assessment^36^(1 = highest predicted resilience relative to other sites, 6 = lowest predicted resilience). Inset plots show the relative resilience of each site over time as predicted using large-area imagery collected in 2014, 2015, 2017, 2019, and 2021. The predicted resilience of each site fluctuated over time, although certain sites were consistently predicted to have high resilience (Molokini) or low resilience (Wahikuli). Inset map shows Maui (in blue) with respect to the rest of the Hawaiian Islands.

While our resilience predictions showed minor fluctuations through time, certain sites were consistently predicted to have high (e.g., Molokini) or low (e.g., Wahikuli) relative resilience (Fig. 1). The temporal fluctuations we observe are consistent with other sensitivity analyses of resilience assessments, which have found that applying different weighting schemes to resilience indicators disproportionately impacts the rank order of mid-tier sites, whereas high or low resilience sites are predicted more consistently^11,42^. The precision of resilience rankings over time is not entirely unexpected for Maui, since other studies have found high stability in benthic community composition over time across Maui Nui^50^. Indeed, the successional trajectories of our sites occupy unique regions of multidimensional space (Supplementary Fig. 1), which suggests that spatial variability in community composition is greater than temporal variability at the scale of our study. We might expect decreased temporal precision in resilience scores in less stable systems, or if resilience was quantified at a different scale^47^.

Part of the reason certain sites were consistently predicted to have high relative resilience year after year was they had one or two indicators that consistently outperformed all other sites. This was especially true for Molokini, which was characterized by relatively high coral cover and calcifying cover, and Keawakapu, which was characterized by relatively high rugosity and coral diversity (Supplementary Fig. 2). This highlights how outliers in a subset of indicators can dampen the signal of all others, potentially leading to predictions that are not representative of the full ecological dynamics at play. In addition, since some indicators showed more inter-site variability than others, more variable indicators had a disproportionate impact on overall resilience predictions. For example, juvenile coral density and reef builder ratio showed more variability between sites than did rugosity, coral health, and coral diversity (Supplementary Fig. 3). Variability in juvenile coral density was driven by exceptionally low densities at certain sites, including a complete lack of juveniles detected in 2021 at four of the six sites. While this was due in part to our focus on *Pocillopora* juveniles, other assessments of juvenile corals in Hawai’i, including the in situ 2018 assessment at these same sites, have found high spatial and temporal variability in juvenile coral density across multiple taxa (Supplementary Fig. 3)^36,42,51^.

### Low accuracy of resilience assessment predictions

**Fig. 2:**
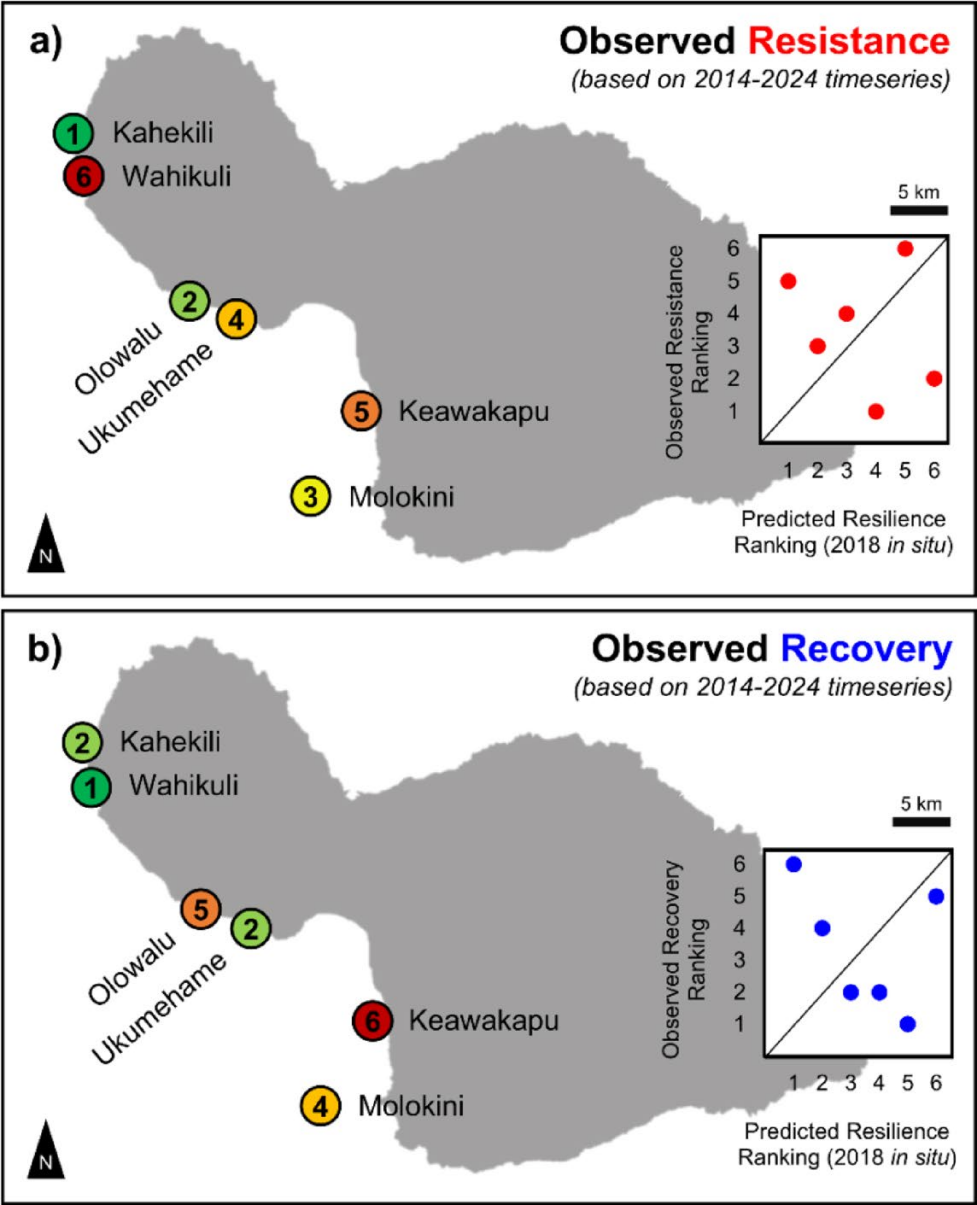
Sites in Maui symbolized by their observed (**a**) resistance and (**b**) recovery over the duration of the timeseries (2014–2024), with 1 being the most resistance or recovery observed, and 6 being the least. Resistance and recovery were quantified in response to the 2015 bleaching event using multi-scale metrics derived from large-area imagery (see Table 2). Inset plots show the lack of alignment between resilience predictions (from the in situ 2018 assessment) and both resistance and recovery. If resilience predictions were 100% accurate, all points would fall along the black 1:1 line (e.g., the site predicted to be most resilient would also be the site with the most resistance and recovery). Scatterplots of resilience predictions from other years are shown in Supplementary Fig. 4.

**Fig. 3:**
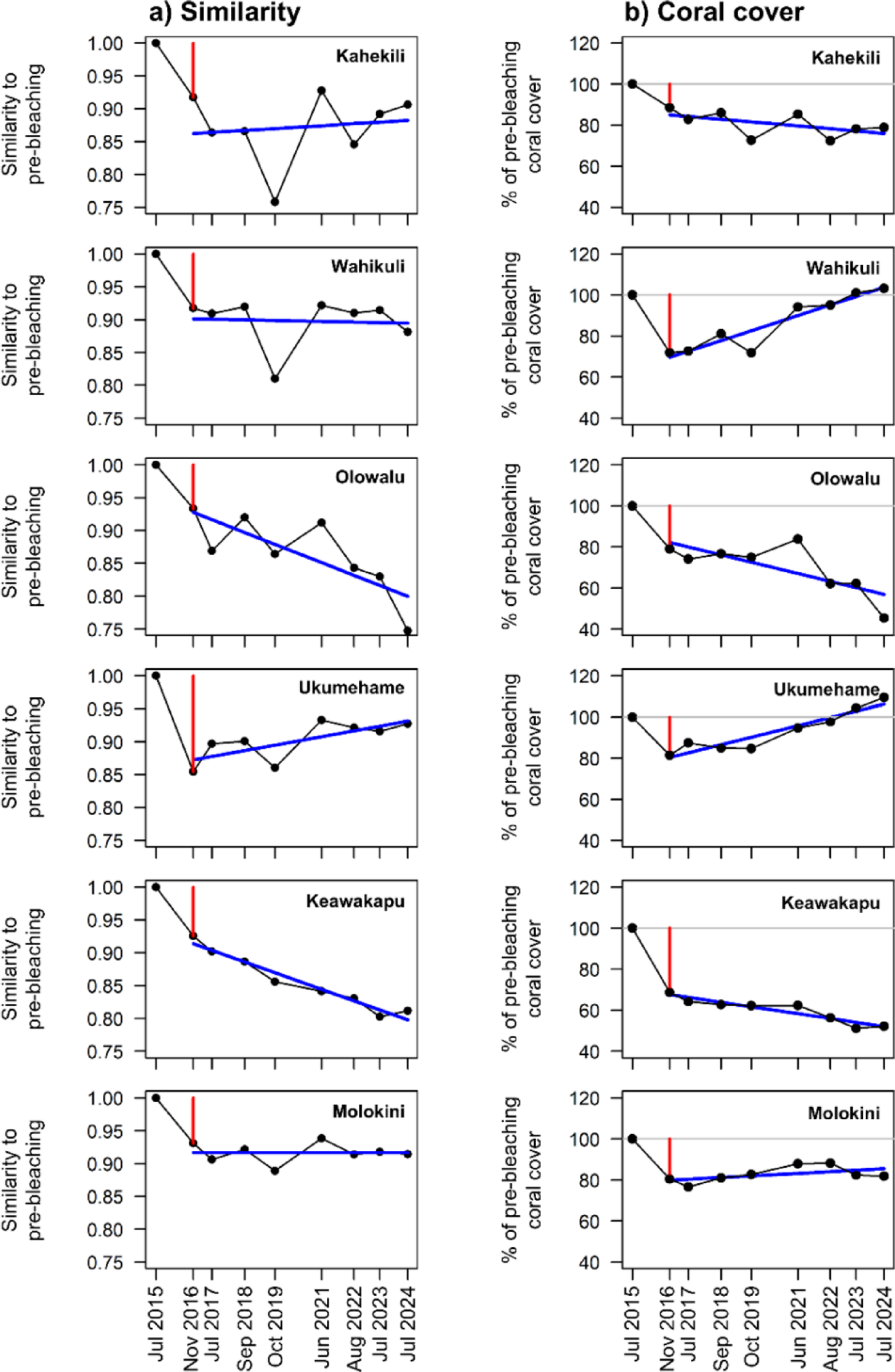
Community-scale indicators of resistance (red) and recovery (blue) with respect to the 2015 bleaching event for six sites in leeward Maui. Graphs show (**a**) the Bray-Curtis similarity between the pre-bleaching benthic community and the benthic community in subsequent years, and (**b**) coral cover as the percent of pre-bleaching cover in July 2015. Resistance to disturbance, denoted by the red vertical line, is quantified as (**a**) the similarity between the pre-bleaching (July 2015) and post-bleaching (November 2016) community, and (**b**) the change in coral cover from July 2015 to November 2016. Low similarity and large declines in coral cover indicate that a site has low resistance. Recovery, denoted by the blue line of best fit, is quantified as the annual rate of change in (**a**) similarity and (**b**) coral cover. A positive slope indicates that a site is regaining coral cover and returning toward its initial community composition, while a negative slope indicates continued change from the pre-bleaching community.

We found little correlation between predicted resilience and observed patterns of resistance or recovery for our sites in leeward Maui (Fig. 2, Supplementary Fig. 4). Keawakapu showed the biggest deviation from expectations: this site was predicted to be the most resilient by the in situ assessment, but demonstrated the second lowest resistance and lowest recovery of all sites over our timeseries, driven by high partial mortality and low coral survivorship (Supplementary Fig. 2). Coral cover dropped from a pre-bleaching high of 55.1% to 37.8% one year post-bleaching and has continued to decline, reaching 28.8% in 2024 (Fig. 3), representing the largest decline across our sites. Notably, this site appears to be experiencing a slow directional shift in benthic community composition towards encrusting algae that may be unrelated to the 2015 bleaching event (Supplementary Fig. 1), which highlights the challenge of predicting ecosystem response to acute disturbances amidst a shifting baseline of chronic degradation^52,53^.

**Fig. 4:**
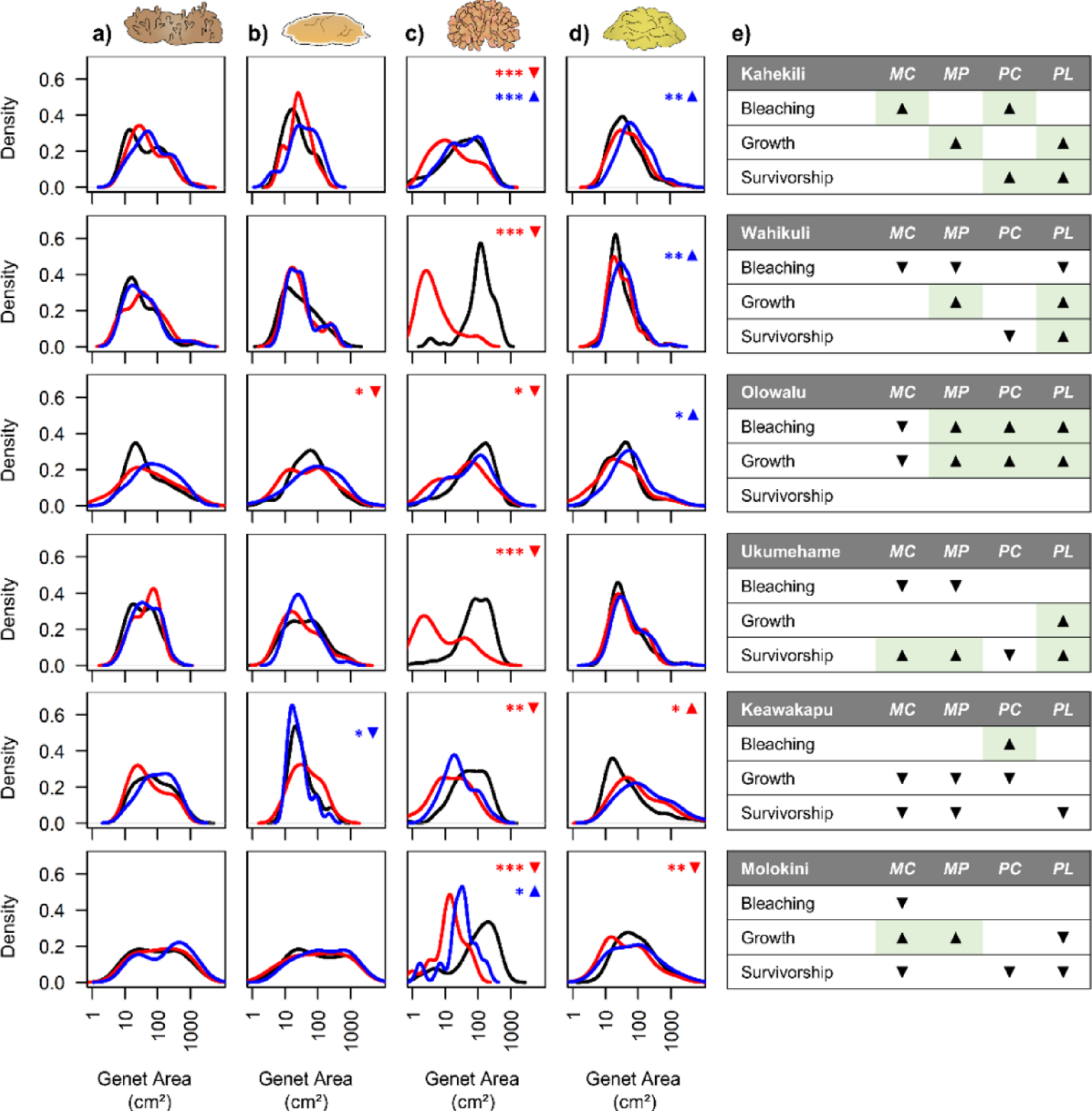
Colony-scale indicators of resistance and recovery from the 2015 bleaching event for six sites in leeward Maui. For each site, coral size frequency distributions are shown for (**a**) *Montipora capitata*, (**b**) *Montipora patula*, (**c**) *Pocillopora* spp., and (**d**) *Porites lobata* in 2014 (black, pre-bleaching), 2017 (red, 2 years post-bleaching), and 2021 (blue, 6 years post-bleaching). Asterisks denote significant change in a population’s size frequency distribution from 2014 to 2017 (red) or 2017 to 2021 (blue), while arrows indicate the direction of the change (see Supplementary Table 2 for p values). Size frequency distributions for *Pocillopora* in 2021 are omitted for Ukumehame and Wahikuli due to a small sample size (*n* < 10). (**e**) Patterns of bleaching susceptibility, growth, and survivorship are shown for each taxon at each site, adapted from McCarthy et al. 2024^46^. Black arrows indicate post-hoc groupings with significantly higher or lower rates of thermal tolerance/acclimatization, growth, or survivorship relative to the other six sites in this study, while blank cells indicate no significant difference. Abbreviations represent taxa in a-d: MC = *M. capitata*, MP = *M. patula*, PC = *Pocillopora*, and PL = *P. lobata*.

In contrast, some sites predicted to have low resilience demonstrated strong recovery potential. This was true for Wahikuli and Ukumehame, which experienced sizeable declines in coral cover after the 2015 bleaching event but saw coral cover rebound to exceed pre-bleaching levels by 2024 (Fig. 3b). Both of these sites share similar community composition (Supplementary Fig. 1) and resilience indicator scores (Supplementary Fig. 2), and demonstrated high survivorship of the stress tolerant massive coral *Porites lobata* over our timeseries (Fig. 4). Other reefs dominated by *P. lobata* in the broader Maui Nui region have also shown recovery potential in recent years, more so than sites dominated by branching *Porites compressa* or encrusting *Montipora* corals^50^. Taken together, this suggests that abundance of *P. lobata* might be a useful indicator of recovery potential for reefs in Maui.

Other sites showed higher resistance than predicted. At Kahekili and Olowalu, resistance was driven by a high proportion of thermally tolerant and acclimatized corals, as well as high coral survivorship at Kahekili (Fig. 4e). This is notable because Kahekili has struggled with wastewater inputs and periodic algae blooms for decades^54^, which negatively impact local coral populations^55^. In response to these stressors, the state created the Kahekili Herbivorous Fisheries Management Area in 2009 to promote herbivory and control algae growth^56^. This management could help explain the resistance we observe. Still, even with management to support herbivorous fish, Kahekili still has higher abundance of macroalgae and cyanobacteria than elsewhere in Maui^57^, factors that have been shown to be negatively associated with resistance and recovery^22,58^.

### What is driving the disconnect between resilience assessment predictions and observed patterns of resistance and recovery?

This is not the first study to document a resilience assessment with low predictive accuracy. A retrospective analysis of a resilience assessment from west Hawai’i island found no correlation between resilience predictions and reef resistance (quantified as the change in coral abundance from 2015 to 2016, as well as bleaching prevalence) or recovery (quantified as the change in coral abundance from 2016 to 2019)^42^. Using multiple metrics of resistance and recovery (Table 2), our results corroborate those findings and highlight the limitations of the resilience assessment methodology. Here we discuss three potential explanations for the lack of concordance between resilience predictions and observed patterns of resistance and recovery: (1) resistance and recovery need to be modeled separately, (2) different or additional resilience indicators need to be used, and (3) our current resilience indicators need to be interpreted differently.

To the first point, an ecosystem’s capacity to resist and recover from disturbance are controlled by fundamentally different processes^26,59^. For example, stress-tolerant taxa within a community are often better positioned to survive disturbance events, while community recovery may initially be driven by faster growing or more fecund taxa^60,61^. This disconnect was apparent for our sites in leeward Maui, as site rankings of resistance and recovery were not correlated (*r* = -0.109). Choosing indicators that are functionally linked to either resistance or recovery could help improve future resilience assessments^14^. While some studies have taken this approach, it is still common practice to combine separate resistance and recovery indicators to generate a single resilience score^62,63^. We argue that collapsing resistance and recovery into a single metric of resilience reduces assessment interpretability and utility for informing management. Modeling resistance and recovery separately could help to differentiate sites like Olowalu (high resistance, low recovery) and Wahikuli (low resistance, high recovery), which likely require different management interventions.

Second, different indicators may have produced more accurate resilience predictions. There is robust empirical support for the resilience indicators used here^14,27,64^, which have been applied widely in other resilience assessments^43^. Maynard et al. 2019^36^ customized indicator selection for their 2018 in situ assessment to reflect local context, eschewing some commonly used indicators (i.e., macroalgae abundance, thermal variability) because they displayed little meaningful variation across their sites in leeward Maui. Still, even the empirically supported indicators used here poorly predicted both resistance and recovery. While coral cover and coral health were moderately correlated with resistance, no indicators were positively correlated with recovery (Supplementary Fig. 5). Other indicators not measured here, such as herbivorous fish biomass, coral predation scars, or the abundance of thermally tolerant corals, may have performed better. In general, indicators associated with reef function, such as recruitment or herbivory, have been shown to better predict community response to both disturbance and management than indicators without a functional connection^65,66^. However, where resilience is controlled by a small number of influential processes^14^, extraneous indicators may diminish the signal of meaningful ones^28,37^. Including multiple indicators of the same process can bias resilience predictions toward that process, and as such, others have argued against including indicators of anthropogenic stressors (i.e., sedimentation, nutrient input) since the impact of these stressors should be already reflected by ecological indicators^37^.

Finally, the poor predictive accuracy of this resilience assessment could stem from our interpretation of the resilience indicators used here. Assigning a value judgement to indicators, where high normalized values denote low vulnerability to disturbance, assumes a unidirectional relationship between reef processes and resilience that may be overly simplistic^15,66,67^. Shifts in species composition and competitive dynamics caused by chronic degradation can decouple expected relationships between environmental drivers and reef performance^53^. In some instances, human-impacted reefs have been shown to be more stable^50,68^ or exhibit faster recovery following disturbance^25,69^ compared to more pristine reefs. The reasons for this disconnect are multifaceted, and can be partially attributed to the effect of disturbance on benthic competition^65^ and selection for stress-tolerant individuals over time^61^. In this study, we found a moderate negative correlation between recovery and several resilience indicators (rugosity, coral diversity, coral health, and reef builder ratio; Supplementary Fig. 5), which suggests that reefs exhibiting signs of degradation are actually showing greater recovery capacity than more pristine reefs. Stress tolerant corals like *P. lobata* may have a competitive advantage at sites with high human impact, and may be better able to persist and recover following disturbance^70^. This notion of increased resilience with increasing degradation has been proposed previously for coral reefs^59^ and other ecosystems^71^. Importantly, we do not suggest that more degraded communities are preferable to pristine ones: while degraded communities might exhibit more stability, the chronic removal of sensitive taxa represents a loss of functional diversity that can diminish provisioning of ecosystem services^52^. Rather, we suggest that degraded communities exhibit potential for resilience and should not be overlooked in a management context^72^.

### Management implications

Resilience-based management aims to (1) identify “resilient” reefs with low vulnerability to climate change so that more resilient locations can be prioritized for management; and (2) inform site-specific management interventions to ensure the persistence of priority reefs^11,12,20^. In regards to the first aim, our results suggest that resilience assessment predictions should be applied with great caution when prioritizing sites for conservation, given the low predictive accuracy of this and other resilience assessments^42^. In circumstances where resilience assessments are planned (or have already been applied) to prioritize management, we strongly recommend that practitioners continue monitoring functional indicators of resistance and recovery to validate the accuracy of resilience predictions over time.

In regards to the second aim, we maintain that resilience assessments have immense potential to support site-specific management, particularly by identifying the ecological mechanisms responsible for resistance and recovery. For instance, a reef experiencing protracted coral decline (such as Keawakapu) could be suffering from suppressed herbivory, elevated nutrients, or outbreaks of coral predators, each of which would prompt a different management response (fisheries regulations, land-based pollution remediation, and predator removal, respectively). Functional indicators of resistance and recovery can help managers identify the right interventions for a given site^73,74^ and distinguish between manageable vs. unmanageable stressors^18,67^. Resilience assessments have been used extensively in this regard. A global analysis of coral reef resilience assessments found that more than half resulted in management actions^12^, including marine spatial planning^31,34,39^, fisheries management^32,38^, and restoration^40^. When ecological indicators are combined with social indicators, resilience assessments can be used to manage reefs as interconnected socio-ecological systems^17^.

To reconcile the two aims of resilience assessments, we suggest managers rely primarily on input from local communities to prioritize specific locations for management. Once these locations have been identified, managers can work with communities to develop custom management strategies informed by site-level indicators of resistance and recovery, doing so in a manner that is supportive and collaborative rather than prescriptive. This distinction is worth emphasizing, as local consultation is not currently common practice for resilience assessments. As of 2021, only 6% of coral reef resilience assessments explicitly mentioned efforts to raise awareness among local stakeholders, and a lack of political will and community buy-in have emerged as major barriers to implementing resilience assessment findings^12^. Centering community input in resilience assessments has the potential to improve conservation efficacy and efficiency by harnessing traditional knowledge and improving distributive and procedural justice in management processes, which in turn solidifies local support for conservation^1,74,75^. Such an approach would more equitable and legitimate than an approach based on ecological indicators alone^74,76,77^. This will require more intentional engagement of local stakeholders at multiple steps in the management process, as has been recommended previously^12,35^.

### Limitations and future directions

This study represents the first time large-area imagery has been applied to conduct a resilience assessment, and is one of the only studies to evaluate resilience assessment precision across multiple years and methodologies^37,42^. Still, it is worth considering several limitations to the results presented here. First, our time series does not allow us to characterize pre-bleaching trends associated with other stressors on the ecosystems due to limited observations prior to the bleaching event. As such, some amount of resistance and recovery could be misattributed features of longer-term unobserved changes. Next, we were only able to consider the resilience of a small number of sites (*n* = 6) due to limited spatial overlap between our large-area imagery timeseries and the in situ 2018 resilience assessment. Even with this limited sample size, the amount of effort required to quantify resilience indicators and resistance and recovery metrics was considerable. Our analysis required us to assign taxonomic IDs to > 148,000 stratified random points, delineate and assess the health of > 15,000 patches of live coral tissue, and visually survey > 4000m^2^ of substrate for juvenile corals. AI-assisted image analysis and coral colony segmentation are active areas of research with the potential to speed ecological data extraction from large-area imagery in the near future^78,79^. This automation, combined with longer timeseries, will help researchers and managers collect time-integrated metrics of reef resistance and recovery (i.e., coral growth, survivorship, size frequency distributions, etc.) more quickly and across larger scales^47^.

Another limitation was our inability to obtain fish survey data for our sites. Herbivory regulates the competitive dynamics between corals and algae^80^ and is hypothesized to be one of the most important drivers of reef resilience^14,23,35,66^. While we could have used large-area imagery to quantify the density of other important herbivores, namely urchins, the functional role of herbivorous fish and urchins are not necessarily analogous, and herbivory by reef fish and urchins have been shown to impact benthic community composition in divergent ways^22,81^. To avoid conflating the functional role of different herbivores, we chose to omit herbivore biomass as a resilience indicator in our assessments. We recalculated resilience rankings from Maynard et al. 2019^36^ without herbivore biomass, and found that they were qualitatively similar for our six sites. Thus, it is unlikely that our failure to include fish biomass explains the low predictive accuracy of resilience assessments for our sites in leeward Maui. Still, we recommend that future resilience assessments include indicators of herbivory if at all possible.

A final consideration is whether the scale of our study is sufficient to observe recovery dynamics^42,47,82^. Based on recovery rates observed elsewhere in the Pacific, annual monitoring over nearly a decade should be sufficient to resolve initial trajectories of community recovery (or lack thereof) in response to bleaching^69,83,84^. However, transient dynamics in community composition can play out over decades, clouding the recovery dynamics of otherwise resilient reefs^82^. Thus, only additional monitoring can confirm whether the trends we observed will continue. Similarly, other resilience assessments have found high levels of spatial variability in resilience scores^11,28,35^, and it is likely that our six sites do not capture the full variability of reef resilience in leeward Maui. We encourage readers to refer to related research on benthic community successional dynamics across the Maui Nui region^50^ to help contextualize the resilience assessment results presented here.

## Conclusions

Using a large-area imagery timeseries and standard indicators of reef resilience, we found that resilience assessments are capable of producing consistent site-level predictions of resilience over time. However, these predictions did not accurately reflect trends in resistance and recovery for our sites in leeward Maui. While indicators of resistance and recovery have value for informing site-specific management at reefs deemed to be important by local communities, our work suggests that quantitative resilience predictions may not be appropriate as a guiding factor in marine spatial planning. Care should be taken to select indicators that are appropriate for a given context and thought to be functionally related to reef processes^14,15,66^. Critically, managers should take steps to make resilience assessments transparent and accessible to the general public, following a participatory process that treats resilience predictions as a starting point for community-driven prioritization, rather than an endpoint for decision making^12,35^.

Given the demonstrated limitations of resilience assessments, it’s worth considering whether resilience is a suitable guiding principle for management decision making. Concepts underpinning resilience-based management should be re-examined, namely the assumption that more pristine sites are less vulnerable to disturbance^59^. More research is needed to develop process-based indicators of reef function^12,24,66,82^ and determine whether these functions are related to resistance or to recovery^20,85^. Process-based metrics of demography, such as size frequency distributions, growth rates, and survivorship, may prove particularly useful for identifying mechanistic drivers of resistance and recovery at a population level^14,49,86^. Long-term monitoring, supported by conservation technology like large-area imaging, will be useful in this regard as it will enable novel metrics to be quantified across multiple scales in space and time.

Beyond resilience assessments, our findings highlight the limitations of using combined sets of ecological indicators to make simple and accurate predictions. This is notable, given the international push to develop standardized indicators for biodiversity credits and corporate nature-related risk disclosures^6,7,87^. As a general rule, we recommend these efforts focus on indicators that are ecologically relevant, responsive, directional, simple to understand, and measurable with local resources, and provide explicit flexibility to select indicators with relevance to local context and management goals. Importantly, indicators should be tracked over time to confirm predictions of resilience, biodiversity, or ecosystem condition. Best-practices for selecting ecological indicators^3,8–10^ should be used as a starting point for developing sets of indicators, which are best implemented in conjunction with local stakeholders.

## Methods

### In situ 2018 resilience assessment

In March 2018, researchers from the Nature Conservancy and Hawaii Division of Aquatic Resources surveyed seven indicators of reef resilience (Table 1) at 31 shallow (3–7 m) and 20 deep (8–13 m) sites in leeward Maui (hereafter referred to as the in situ survey). In situ survey methods are described in detail in Maynard et al. 2019^36^. Briefly, divers assessed coral cover, coral diversity, and the ratio of calcifying to fleshy benthic cover (reef builder ratio) using 20 randomly selected 0.8 × 0.6 m photoquadrats collected along three 25 m transects. They also conducted visual surveys of coral health and juvenile coral density within twelve 0.25m^2^ quadrats, assessed linear rugosity using a 10 m brass chain, and estimated the length and biomass of herbivorous fish within three 25 × 5 m belt transects. The seven resilience indicators were normalized and used to calculate each site’s relative resilience. The final result was a ranking of sites based on their predicted resilience, which informed the development of site-specific management recommendations^36^.

### Large-area imagery timeseries

Researchers from the Scripps Institution of Oceanography began using large-area imagery to monitor coral reefs in leeward Maui in 2014, and have surveyed the same fixed 10 × 10 m sites annually, with the exception of 2020. Sites were surveyed twice in 2015, once in July (pre-bleaching) and once in November, coinciding with the peak of a major bleaching event^88^. Six large-area imagery sites are located closely to or directly overlap with sites used in the in situ 2018 resilience assessment (Kahekili, Wahikuli, Olowalu, Ukumehame, Keawakapu, and Molokini; Fig. 1; Supplementary Table 1): Those hardbottom forereef sites are the focus of this study and range between 4.5 and 10 m depth.

To conduct large-area imagery surveys, divers relocated fixed monitoring sites with the aid of GPS coordinates, permanent stainless-steel stakes or eye-bolts, and a 2D photomosaic “site map” printed on underwater paper. Once the site was located, divers placed six calibration tiles and four floats to mark the edges of the fixed 10 × 10 m site, and placed four 0.5 m scale bars within the site. One diver measured the depth of each calibration tile and, prior to 2016, the distance between calibration tiles in lieu of placing scale bars. The other diver swam approximately 1.5 m above the substrate in a gridded pattern holding a custom rig equipped with two Nikon D7000 SLR cameras (except in 2023 and 2024, where Nikon D780 SLRs were used) in Ikelite underwater housings. One camera was equipped with a wide-angle lens (18 mm for 2014–2022, 24 mm for 2023–2024) and the other was equipped with a telephoto lens (55 mm for 2014–2022, 60 mm for 2023–2024). Focal lengths were adjusted in 2023 to ensure that the spatial footprint of each photo was consistent between the D7000 and D780 cameras. Each camera used a 1 s interval timer to take approximately 2500 photos (5,000 total photos per site). The camera diver swam several meters beyond the 10 × 10 m site boundary to ensure a buffer zone of imagery was collected. In water image collection methods are described in further detail in Pedersen et al. 2025^89^.

Once imagery was collected, researchers used the software *Agisoft Metashape* (St. Petersburg, Russia) to align the 5,000 underwater images (hereafter referred to as raw images) and build a dense point cloud reconstruction of the benthos (hereafter referred to as the 3D model). Once the 3D model was constructed, researchers loaded it into *Viscore*, a custom data visualization and ecological analysis software developed at UC San Diego^90^. Using Viscore, researchers entered depth and scaling metadata collected in the field, color corrected imagery, and manually aligned 3D models of the same site collected in different years^89^. These aligned timeseries of 3D models form the basis of ecological analysis conducted in this study.

### Assessing resilience indicators using large-area imagery

For each site in each timepoint, we used our large-area imagery timeseries to assess six of the seven resilience indicators used by Maynard et al. 2019^36^. Large-area imagery has been shown to be reliable source of ecological data on coral cover, health, and demography, as well as 3D structural metrics, with accuracy comparable to or exceeding that of in situ surveys^91,92^. However, because large-area imagery only reconstructs static features of the benthos, we were not able to assess herbivorous fish biomass using our timeseries. To achieve consistency across datasets, we omitted herbivorous fish biomass as a resilience indicator and re-calculated resilience scores from Maynard et al. 2019^36^ using the remaining six indicators: percent coral cover, reef builder ratio, coral diversity, juvenile coral density, coral health, and linear rugosity (Table 1).

We calculated the percent cover of benthic taxa using Viscore’s virtual point intercept tool (VPI)^89^, which places a user-defined number of stratified random points (here 2,500) within a single large quadrat (here 10 × 10 m) in the coral reef 3D model. The user has access to the raw imagery that underlies the 3D model, which allows each VPI point to be viewed from multiple angles before a taxonomic ID is assigned. A single researcher with experience in Hawaiian benthic taxonomy (OM) identified the benthos under each VPI point to the lowest possible taxonomic resolution, and taxa were later assigned to one of 15 functional groups (seven coral taxa, six algal functional groups, “other” sessile invertebrates (i.e., tunicates, sponges, or zoanthids), or sand; Supplementary Fig. 6). We used VPI data to calculate three of our resilience indicators: percent coral cover, coral diversity (Simpsons diversity), and reef builder ratio (ratio of the percent cover of corals and calcifying algae to the percent cover of fleshy algae and other sessile invertebrates).

For juvenile coral surveys, we counted all *Pocillopora* colonies < 5 cm in diameter within a 12 × 12 m plot, only including corals that had not been detected in a previous timestep. We chose not to include *Montipora* and *Porites* due to the difficulty of distinguishing true juveniles from partial mortality of larger colonies via benthic imagery. *Montipora* and *Porites* commonly undergo fission and fusion, which can make it difficult to distinguish colony boundaries after years of partial mortality and regrowth^46^. *Pocillopora* have a more discrete shape, less partial mortality, and easily identifiable juveniles. While the 2018 in situ survey included juvenile *Montipora* and *Porites*, in situ coral recruit surveys are capable of achieving higher resolution than solely image-based approaches^93^. Focusing solely on *Pocillopora* in the large-area imagery surveys allowed us to have confidence in our image-based assessments of juvenile coral density.

To assess coral health, we surveyed corals for signs of severe bleaching, algal overgrowth, and coral disease. Corals that exhibited one or more of these conditions were considered to have compromised health. We surveyed each coral visually using the raw images that underly the 3D model. Our ability to access to multiple images of any given coral allowed us to distinguish apparent signs of compromised health (i.e., discoloration) that were artifacts of poor image quality or overexposure. We assessed the health of individual corals in four taxa (*Montipora capitata*, *Montipora patula*, *Pocillopora* spp., and *P. lobata*) that we also tracked each coral for colony-scale analyses of resistance and recovery (see Sect. 5.2). We calculated the prevalence of compromised health for each taxon and then calculated a single rate of compromised health for each site, weighting the four taxa equally. Maynard et al. 2019^36^ assessed the percent of colonies with signs of compromised health in situ and then inverted the normalized value so that high values would denote high resilience. Here, we simply used the percent of colonies without signs of compromised health as our resilience indicator, as has been done elsewhere^11^, and recalculated values from Maynard et al. 2019^36^ accordingly.

Finally, we assessed linear rugosity using Viscore’s virtual profile gauge tool^94^, which measures the depth of the reef contour at a fixed interval (here every 1 cm) along a series of linear transects (here 10 m long). We calculated linear rugosity as the distance traversed by each reef contour divided by the linear distance traversed by each transect. For linear rugosity, a value of 1 corresponds to a completely flat surface, with increasing large values denoting increasingly rough surfaces with greater structural complexity. We measured linear rugosity in the alongshore direction using 99 transects, each spaced 10 cm apart, to cover the same spatial footprint as the 100m^2^ quadrat used to assess percent cover.

### Using indicators to predict resilience

We predicted site-level resilience based on six indicators (Table 1) following the approach of Maynard et al. 2019^36^, which is consistent with other resilience assessments^11,18,35,42^. We predicted resilience separately for each year where data was available for all indicators. This corresponded to five years of large-area imagery data (2014, 2015, 2017, 2019, and 2021) and one year of in situ data (2018). For each year, we normalized each resilience indicator by dividing by its maximum value. Following this approach, the “best performing” site for a given indicator would have a normalized value of 1, and normalized values of lower performing sites would represent a decimal percentage relative to the best performing site. We calculated the unweighted average of the six normalized indicators to generate a resilience score for each site. Finally, we normalized resilience scores to calculate each site’s relative resilience, where the site predicted to be most resilient had a value of 1 (Appendix 1). To examine the temporal variability of resilience predictions and the relative influence of different resilience indicators, we performed a canonical analysis of principal coordinates (CAP; Supplementary Fig. 2) using the Euclidean distance matrix of normalized resilience indicators for all sites in all years^35,36^.

### Quantifying observed patterns of resistance and recovery

#### Community-scale indicators of resistance and recovery

Following the approach of McCarthy et al. 2025^50^, we quantified benthic community composition using multivariate data collected from 3D coral reef models. Multivariate data consisted of percent cover of benthic taxa (7 coral taxa, 6 algal functional groups, other sessile invertebrates, and sand; Supplementary Fig. 6), structural complexity (linear rugosity at 1 cm and 50 cm resolution and fractal dimension), and landscape heterogeneity (proportion of unlike adjacencies, derived from percent cover data). Using this multivariate data for our six focal sites across ten years of sampling, we calculated the Bray-Curtis dissimilarity matrix and used non-metric multidimensional scaling (NMDS) to visualize successional trajectories for each site (Supplementary Fig. 1). We included benthic community data from 30 other forereef sites in Maui Nui (surveyed in 2021) in our NMDS so that the successional trajectories of our six focal sites could be interpreted within a broader regional context.

To quantify community-scale patterns of resistance and recovery for each site (Table 2), we calculated the similarity in community composition between the last pre-bleaching survey (July 2015) and each post-bleaching survey (November 2016 through July 2024). Similarity is the inverse of dissimilarity, which we calculated using the Bray-Curtis index via the vegan package in R^95^. We quantified resistance as the similarity between the last pre-bleaching survey (July 2015) and the first post-bleaching survey (November 2016), with high similarity indicating high resistance to bleaching impacts^72,96^. To quantify recovery, we calculated the slope of the linear regression through similarity values from 2016 to 2024, where a positive slope indicated that community composition became more similar to the pre-disturbance community over time^97^. We also quantified resistance and recovery for each site based on changes in coral cover over time, since percent coral cover is a widely used metric of coral reef health with direct relevance to resource managers^14,66^. Here we quantified resistance as the percent of pre-bleaching coral cover that had been lost one-year post-bleaching. For recovery, we calculated the annual rate of coral cover change from 2016 to 2024, and interpreted a positive slope as an indicator of coral recovery^25,69^. Finally, to account for minor differences in thermal exposure between sites^46^, we standardized rates of resistance and recovery by the maximum degree heating weeks each site experienced in 2015.

#### Colony-scale indicators of resistance and recovery

For our colony scale analysis, we quantified the size-frequency distribution of four coral taxa (*M. capitata*, *M. patula*, *Pocillopora* spp., and *P. lobata*) to track demographic shifts in coral populations in response to the 2015 bleaching event. To do this, we traced the boundaries of corals using 2D orthoprojections of 3D coral reef models following the approach of Rodriguez et al. 2021^86^. We used the freeware semantic segmentation platform TagLab^78^ to trace coral boundaries on the orthoprojection and link corals through time that represented the same individual. To ensure accuracy, we viewed the original imagery used to generate the 3D model alongside the orthoprojection to clarify any questions about coral boundaries. Since corals can split into multiple independent ramets via partial mortality (fission), which can later regrow back into a single colony (fusion), we considered all patches of live coral tissue linked in time via fission and fusion to represent a single “genet” and calculated size frequency distributions for coral genets, rather than coral patches, as has been done elsewhere^46,86^.

To achieve an appropriate sample size of genets, we traced corals within 10 randomly placed 0.5m^2^ quadrats. We increased the number of quadrats for less abundant taxa until we reached a minimum sample size of 40 genets or had placed 25 quadrats. We traced all genets that had at least one patch of live coral tissue > 5 cm diameter with a centroid inside a quadrat. If a genet was traced in one year, we searched for it in other years and traced all associated patches, even if they were smaller than our 5 cm diameter cutoff or located outside of a quadrat. We summed the planar area of interconnected patches to calculate the planar area (cm^2^) of each genet in each year, and then log transformed the planar area to generate a size frequency distribution of coral genets for each taxa at each site^86^. We used 2-sample Kolmogorov-Smirnov (K-S) tests to identify significant shifts in size frequency distributions between 2014 and 2017 and 2017–2021 for each taxon at each site. We interpreted a significant decline in coral size-frequency from 2014 to 2017 as evidence of partial mortality and low resistance to bleaching, and considered a significant increase in coral size frequency from 2017 to 2021 as evidence of recovery (Table 2). In addition to size frequency distributions, we utilized rates of bleaching susceptibility, survivorship, and growth documented by McCarthy et al. 2024^46^ at these same sites. We considered sites with higher proportions of thermally tolerant or acclimatized corals and sites with significantly higher survivorship to have higher resistance, and sites with significantly higher growth rates to have higher recovery (Table 2).

#### Ranking sites based on community- and colony-scale patterns of resistance and recovery

We compiled our disparate community and colony scale metrics into a composite ranking of resistance (Fig. 2a) and recovery (Fig. 2b). For each metric, we assigned the best performing sites a value of 1 and the worst performing sites a value of -1 (Appendix 2). For community scale metrics, we considered sites > 1 standard deviation from the mean (for resistance) or from 0 (for recovery) to be best (+ 1 SD) or worst (-1 SD) performing. For colony scale metrics, we considered sites with significantly higher rates of thermal tolerance, survivorship, growth, or a significant size frequency shift back toward their pre-disturbance distribution to be best performing, and those with significantly higher rates of bleaching, mortality, shrinkage, or a significant size frequency shift away from their pre-disturbance distribution to be worst performing. Sites that did not qualify as best or worst performing for a given metric were coded as 0. Once all metrics were coded as -1, 0, or 1, we calculated the mean value of resistance and recovery for each site, weighting of community and colony scale metrics so that each scale accounted for 50% of the final resistance and recovery ranking (Appendix 2). Finally, we regressed site resistance and recovery rankings against predicted resilience rankings for each year that a resilience assessment was conducted (Supplementary Fig. 4) to evaluate the predictive accuracy of resilience assessments for our six sites in leeward Maui.

## Supplementary Information

Supplementary Information 1.

Supplementary Information 2.

Supplementary Information 3.

Supplementary Information 4.

## Data Availability

Data is provided within the supplementary information files.
