# Supplementary material for: Evaluating the predictive capacity of coral reef resilience assessments
Source: Sci Rep. 2026 May 30;16:24804. doi: 10.1038/s41598-026-52791-2 (PMC13457859; doi:10.1038/s41598-026-52791-2)
Supplement: Supplementary file 1 [file 41598_2026_52791_MOESM1_ESM.docx]

**Supplementary Materials for *Evaluating the predictive capacity of coral reef resilience assessments***

Orion S. McCarthy*^1^, Eric Conklin^2^, Katharine Ricke^1,3^, Russell T. Sparks^4^, and Jennifer E. Smith^1^

1. Scripps Institution of Oceanography, UC San Diego, La Jolla, CA 92037
2. The Nature Conservancy – Hawai'i and Palmyra, Honolulu, HI 96817
3. School of Global Policy and Strategy, UC San Diego, La Jolla, CA 92037
4. Maui Division of Aquatic Resources, Department of Land and Natural Resources, Wailuku, HI 96793

**Supplementary Fig. 1.** Non-metric multidimensional scaling ordination of benthic community composition at six sites in leeward Maui. Each color corresponds to a different site, while the number inside each point corresponds to the year of the survey. Surveys connected in a chronological sequence to form a successional trajectory. Grey dots represent additional sites from the Maui Nui region (surveyed in 2021) from McCarthy et al. 2025. Abbreviations correspond to the following benthic variables: Mon.cap = *Montipora capitata*; Mon.pat = *Montipora patula*; Por.com = *Porites compressa*; Por.lob = *Porites lobata*; Poc = *Pocillopora* spp.; Pav = *Pavona* spp.; Oth.cor = other coral taxa; CCA = crustose coralline algae; Turf = turf algae; Cyano = cyanobacteria; Fl.mac = fleshy erect macroalgae; Lob.Peys = fleshy encrusting macroalgae; Sand = sand; r.1cm = linear rugosity measured at 1cm resolution; r.50cm = linear rugosity measured at 50cm resolution; frac = fractal dimension; hetero = landscape heterogeneity.

**Supplementary Fig. 2.** Canonical Analysis of Principal Coordinates (CAP) ordination showing the relative contribution of the six resilience indicators (arrows) to resilience scores. Sites are color-coded, and the number inside each point corresponds to the year of the survey. Ellipses show the standard deviation of resilience scores for each site. Point shape indicates the organization that conducted the resilience assessment (square for *in situ* surveys conducted by the Nature Conservancy (TNC), circle for large-area imagery surveys conducted by the Scripps Institution of Oceanography (SIO)). Resilience indicator abbreviations are as follows: CC = coral cover, RBR = reef builder ratio, CDiv = coral diversity, Hlth = coral health, Juv = juvenile coral density, Rugo = linear rugosity.

**Supplementary Fig. 3**. Normalized values for six resilience indicators. Boxplot colors denote the year each resilience indicator was assessed. Resilience indicators were assessed at six leeward Maui sites (Kahekili, Keawakapu, Molokini, Olowalu, Ukumehame, and Wahikuli) in six different years (2014, 2015, 2017, 2018, 2019, and 2021).

**Supplementary Fig. 4.** Scatterplots show the relationship between resilience rankings (1 = highest resilience, 6 = lowest resilience) and rankings of A) resistance and B) recovery observed over the timeseries. Resilience assessments were conducted by Scripps Institution of Oceanography (SIO) using data from 2014, 2015, 2017, 2019, and 2021, and by the Nature Conservancy (TNC) in 2018. The C) correlation coefficient and D) R squared value for the relationship between predicted resilience and observed resistance (red) and recovery (blue) are shown over time. The 2015 bleaching event is denoted with a thermometer icon in C) and D).

**Supplementary Fig. 5.** We ranked sites based on their coral cover (CC), coral diversity (Cdiv), *Pocillopora* juvenile density (Juv), coral health (Hlth), reef builder ratio (RBR), and rugosity (Rugo) in each resilience assessment year (2014, 2015, 2017, 2018, 2019, and 2021). Here we visualize the strength of the relationship between each resilience indicator and observed resistance (red) and recovery (blue) based on rank order. The range of A) correlation coefficients and B) R squared values are shown for each indicator across all resilience assessments. Overall, indicators showed low predictive accuracy. Coral cover and coral health were moderately strong predictors of resistance, while no indicators accurately predicted recovery.

**Supplementary Fig. 6.** Benthic community composition at six resilience assessments sites in leeward Maui. Surveys conducted during the 2015 bleaching event are denoted with a red triangle. *Porites lobata* includes closely related massive *Porites* species that we could not confidently differentiate via imagery, such as *Porites lutea*, *Porites lichen*, *Porites brighami*, and *Porites evermani*. Taxa in the “other coral” category had low abundance (< 0.1% cover) and include *Leptastera* spp. and *Fungia* spp.

**Supplementary Fig. 7**. Boxplots illustrate the log-transformed planar area of genets for populations of A) *Montipora capitata*, B) *Montipora patula*, C) *Pocillopora* spp., and D) *Porites lobata* at six resilience sites in five different years. A thermometer icon denotes the 2015 bleaching event. Colors denote sites, from left to right: red = Kahekili; magenta = Wahikuli; turquoise = Olowalu; blue = Ukumehame; gold = Keawakapu; green = Molokini.

**Supplementary Table 1**. The coordinates and depth of resilience sites used in this study. The exact location of sites differed slightly between the original *in situ* resilience assessment (conducted in 2018; see Maynard et al. 2019) and the follow up resilience assessments conducted here (using large-area imagery from Scripps Institution of Oceanography’s long-term monitoring sites). The Nature Conservancy surveyed sites in March 2018. The Scripps Institution of Oceanography surveyed sites in July 2014, July 2015, November 2015, November 2016, July 2017, September 2018, October 2019, June 2021, August 2022, July 2023, and July 2024.

| Site | *The Nature Conservancy*  *(TNC)* | | | *Scripps Institution of Oceanography (SIO)* | | | Distance (m) between  SIO and TNC sites |
| --- | --- | --- | --- | --- | --- | --- | --- |
|  | Latitude | Longitude | Depth (m) | Latitude | Longitude | Depth (m) |  |
| Kahekili | 20.93790 | -156.69372 | 6.5 | 20.93677 | -156.69365 | 5.2 | 126 |
| Wahikuli | 20.90983 | -156.69168 | 3.8 | 20.90983 | -156.69168 | 4.5 | 0 |
| Olowalu | 20.80605 | -156.61222 | 4.3 | 20.80465 | -156.60721 | 9.7 | 542 |
| Ukumehame | 20.79098 | -156.58437 | 9.2 | 20.79098 | -156.58437 | 8.6 | 0 |
| Keawakapu | 20.70482 | -156.45037 | 9.6 | 20.70482 | -156.45036 | 10.0 | 1 |
| Molokini | 20.63148 | -156.49638 | 6.2 | 20.63148 | -156.49658 | 8.7 | 20 |

**Supplementary Table 2.** Results from 2-sample Kolmogorov-Smirnov (K-S) tests. Asterisks indicate significant differences in size frequency distributions between 2014 – 2017 (red) and 2017 – 2021 (blue). K-S tests were not conducted for populations < 10 individuals (*Pocillopora* in 2021 at Ukumehame and Wahikuli).

| Taxa | Site | Number of genets | | | P value | |
| --- | --- | --- | --- | --- | --- | --- |
|  |  | 2014 | 2017 | 2021 | 2014 – 2017 | 2017 – 2021 |
| *Montipora capitata* | Kahekili | 45 | 39 | 29 | 0.234 | 0.293 |
|  | Keawakapu | 52 | 41 | 21 | 0.295 | 0.059 |
|  | Molokini | 53 | 47 | 34 | 0.940 | 0.398 |
|  | Olowalu | 74 | 52 | 40 | 0.628 | 0.200 |
|  | Ukumehame | 76 | 65 | 70 | 0.239 | 0.835 |
|  | Wahikuli | 47 | 25 | 29 | 0.088 | 0.419 |
| *Montipora patula* | Kahekili | 31 | 32 | 21 | 0.165 | 0.386 |
|  | Keawakapu | 76 | 47 | 30 | 0.147 | **0.012 *** |
|  | Molokini | 52 | 47 | 41 | 0.995 | 0.697 |
|  | Olowalu | 78 | 69 | 53 | **0.037 *** | 0.249 |
|  | Ukumehame | 43 | 45 | 44 | 0.541 | 0.569 |
|  | Wahikuli | 42 | 43 | 52 | 0.838 | 0.745 |
| *Pocillopora* | Kahekili | 139 | 115 | 73 | **< 0.001 ***** | **< 0.001 ***** |
|  | Keawakapu | 101 | 52 | 24 | **< 0.001 ***** | 0.143 |
|  | Molokini | 94 | 17 | 13 | **< 0.001 ***** | **0.014 *** |
|  | Olowalu | 136 | 41 | 40 | **0.013 *** | 0.121 |
|  | Ukumehame | 162 | 32 | 7 | **< 0.001 ***** | -- |
|  | Wahikuli | 87 | 15 | 9 | **< 0.001 ***** | -- |
| *Porites lobata* | Kahekili | 123 | 103 | 89 | 0.159 | **0.005 **** |
|  | Keawakapu | 72 | 34 | 31 | **0.042 *** | 0.294 |
|  | Molokini | 81 | 54 | 43 | **0.006 **** | 0.174 |
|  | Olowalu | 63 | 52 | 48 | 0.587 | **0.011 *** |
|  | Ukumehame | 80 | 74 | 76 | 0.921 | 0.306 |
|  | Wahikuli | 161 | 140 | 160 | 0.779 | **0.006 **** |
